# Immunization with neural‐derived peptides increases neurogenesis in rats with chronic spinal cord injury

**DOI:** 10.1111/cns.13368

**Published:** 2020-04-30

**Authors:** Roxana Rodríguez‐Barrera, Adrián Flores‐Romero, Elisa García, Ana Maria Fernández‐Presas, Diego Incontri‐Abraham, Lisset Navarro‐Torres, Julián García‐Sánchez, Juan José Juárez‐Vignon Whaley, Ignacio Madrazo, Antonio Ibarra

**Affiliations:** ^1^ Centro de Investigación en Ciencias de la Salud (CICSA) FCS Universidad Anáhuac México Campus Norte Huixquilucan Mexico; ^2^ Departamento de Microbiología y Parasitología Facultad de Medicina Col. Universidad Nacional Autónoma de México Coyoacan Mexico; ^3^ Proyecto CAMINA A.C Tlalpan Mexico; ^4^ Unidad de Investigación Médica en Enfermedades Neurológicas CMN Siglo XXI IMSS Ciudad de México Mexico

**Keywords:** A91 peptide, INDP, neurogenesis, paraplegia, protective autoimmunity, SCI, chronic stage, motor recovery, sensitive recovery, protective autoimmunity, traumatic brain injury

## Abstract

**Aims:**

Immunization with neural‐derived peptides (INDP) has demonstrated to be a promising therapy to achieve a regenerative effect in the chronic phase of the spinal cord injury (SCI). Nevertheless, INDP‐induced neurogenic effects in the chronic stage of SCI have not been explored.

**Methods and Results:**

In this study, we analyzed the effect of INDP on both motor and sensitive function recovery; afterward, we assessed neurogenesis and determined the production of cytokines (IL‐4, IL‐10, and TNF alpha) and neurotrophic factors (BDNF and GAP‐43). During the chronic stage of SCI, rats subjected to INDP showed a significant increase in both motor and sensitive recovery when compared to the control group. Moreover, we found a significant increase in neurogenesis, mainly at the central canal and at both the dorsal and ventral horns of INDP‐treated animals. Finally, INDP induced significant production of antiinflammatory and regeneration‐associated proteins in the chronic stages of SCI.

**Conclusions:**

These findings suggest that INDP has a neurogenic effect that could improve motor and sensitive recovery in the chronic stage of SCI. Moreover, our results also envision the use of INDP as a possible therapeutic strategy for other trauma‐related disorders like traumatic brain injury.

## 1 INTRODUCTION

Spinal cord injury (SCI) is a severe medical condition generally caused by a traumatic mechanism, such as a contusion, compression, or transection.^1^ SCI usually induces acute and chronic repercussions which depend on both the vertebral level and the severity of the lesion.^2^, ^3^, ^4^

After the primary injury—the one induced by the traumatic event—a significant number of self‐destructive mechanisms is triggered, which further increases tissue damage (secondary injury). These destructive phenomena begin approximately 2 hours after the injury and continue until about 6 months after the lesion.^5^, ^6^ During this period of time, the injury worsens by the action of pro‐inflammatory factors, excitotoxicity, vascular alterations, oxidative stress, and ischemia. These destructive mechanisms show a gradual decrease throughout the whole injury until the chronic stage, where they are almost at its minimum expression; however, there is still a progressive decline in the neurological function.^7^ Therefore, in the chronic phase of injury, therapeutic approaches should be directed to restore more than to protect neural tissue. With this in respect, several strategies have been tested in order to promote neural restoration.^8^

In this context, immunization with neural‐derived peptides (INDP) has shown to induce neural restoration and functional recovery in the chronic stage of injury.^9^, ^10^, ^11^ This therapy induces a microenvironment characterized by a significant decrease in pro‐inflammatory as well as an increase in antiinflammatory cytokines. Likewise, there is an important increase in the production of neurotrophic factors. These conditions have been associated with the regeneration of axons as well as with an improved motor recovery.^10^ Since neurogenesis could be another possible mechanism by which INDP improves functional recovery, it is important to assess the effect of this therapy on the appearance of newly formed neurons in the chronic stages of SCI. In fact, INDP (using copolymer‐1) has already shown to induce neurogenesis after a stroke in experimental models.^12^, ^13^ In addition, INDP could also be a relevant therapy for inducing neurogenesis in other trauma‐related disorders such as traumatic brain injury.

INDP, specifically with A91 peptide, has shown to improve motor performance. This peptide is derived from 87‐99 amino acids of the immunogenic sequence of the myelin basic protein. A91 modulates the inflammatory and autoreactive response observed following SCI. These effects are carried out by inducing a Th2 response, thus promoting an M2 macrophage phenotype and providing a permissive microenvironment for functional recovery.^14^, ^15^, ^16^

The aim of this study was to demonstrate whether INDP, using A91 peptide, induces neurogenesis in the chronic stages of SCI.

## 2 MATERIALS AND METHODS

### 2.1 Ethical considerations

All procedures were carried out in accordance with the National Institutes of Health Guide for the Care and Use of Laboratory Animals and the Mexican Official Norm on Principles of Laboratory Animal Care (NOM 062‐ZOO‐1999). In addition, the Animal Bioethics and Welfare Committee approved all animal procedures (ID: 178544; CSNBTBIBAJ 090812960). All experiments were designed and reported according to the ARRIVE guidelines.

In order to perform euthanasia, animals were previously anesthetized by an intramuscular injection of a mixture of ketamine (50 mg/kg) and xylazine (10 mg/kg).

### 2.2 Study design

The experiment consisted of adult female Sprague Dawley rats weighing between 230 and 250 g, which were randomly distributed into two groups (GraphPad QuickCalcs: http://www.graphpad.com/quickcalcs/): (a) phosphate‐buffered saline (PBS)‐immunized rats and (b) INDP‐immunized rats. The sample size for this experiment was calculated using an alpha of 0.05 and a beta of 0.20. At day 0, all rats were subjected to a moderate SC contusion and then housed for postoperative care. On day 60, half of the rats were immunized with A91 and the other half with PBS. The locomotor function was evaluated starting on day 60 after SCI and thereafter weekly throughout 8 weeks, while the sensitivity was assessed at days 0, 60, and 120. At the end of the study, rats were euthanized, and the SC was quickly extracted for further analysis. We determined the neurogenic effect of INDP at the injured site of the SC using immunofluorescence, as well as the expression of interleukin (IL)‐4, IL‐10, tumor necrosis factor (TNF)α, brain‐derived neurotrophic factor (BDNF), and growth‐associated protein (GAP)‐43 protein levels using enzyme‐linked immunosorbent assay (ELISA). The expression of cytokines and neurotrophic factors was determined three times. For each time, the samples were studied by triplicate.

### 2.3 Spinal cord injury

Animals were anesthetized by an intramuscular injection of a mixture of ketamine (50 mg/kg) (Probiomed) and xylazine (10 mg/kg) (Fort Dodge Laboratories). The skin was opened in layers, and a laminectomy was performed at T9 vertebral level of the SC. Subsequently, a 10‐g rod was dropped onto the SC from a height of 25 mm using the NYU impactor (NYU). Functional recovery of all groups was assessed using the Basso, Beattie, and Bresnahan (BBB) locomotor scale.

### 2.4 Postoperative care

After SCI, animals were housed with food and water ad libitum, and received manual bladder voiding, three times a day for 2 weeks. To avoid infection, enrofloxacin (Marvel) was diluted into their drinking water at an approximate dose of 64 mg/kg/d for 1 week. Animals were carefully monitored for signs of infection, dehydration, or automutilation with appropriate veterinary assistance as needed.

### 2.5 Antigen (A91 peptide)

A91 peptide was derived from the encephalitogenic amino acid sequence 87‐99 of the myelin basic protein (MBP). A nonencephalitogenic analog was obtained by replacing the lysine residue with alanine at position 91. The modified peptide was purchased from Invitrogen Life Technologies. Reverse‐phase HPLC confirmed the purity of the A91 peptide (>95%).

#### 2.5.1 Active immunization

Rats were immunized subcutaneously at the base of the tail with 200 μg of A91 dissolved in PBS (experimental group) or only with PBS (control group). A91 and PBS alone were emulsified in an equal volume of complete Freund's adjuvant (CFA) containing 0.5 mg/ml of *Mycobacterium tuberculosis* (Sigma‐Aldrich). Immunization was performed 60 days after SCI.

### 2.6 Functional recovery evaluation

#### 2.6.1 Assessment of motor recovery

Behavioral recovery was assessed using the BBB open‐field locomotor scale method. Animals were evaluated 60 days after SCI and thereafter weekly throughout 8 weeks. Three blinded observers to the treatment performed evaluations. The average of the three scores was used.

#### 2.6.2 von Frey hair test

The rats were placed in a clear acrylic glass enclosure on an elevated metal mesh floor and allowed to acclimate to the new environment for 15 minutes. The paw‐withdrawal response to non‐noxious mechanical stimuli was recorded using an Electronic von Frey Anesthesiometer (IITC Life Science, Inc). The plantar surface of each hind paw of the rats was stimulated using von Frey plastic filaments perpendicularly, and the maximum pressure required to elicit a response was automatically registered. Three scores for each paw were recorded and averaged. This sensitivity analysis was performed before SCI (0 days) to ensure that the animals showed normal responses, and it was repeated after surgery 60 and 120 days later.

### 2.7 Immunofluorescence

Neurogenesis was evaluated by immunofluorescence using a double stain with anti‐5‐bromo‐2′‐deoxyuridine (BrdU) and doublecortin (Dcx) antibodies. BrdU is a synthetic nucleotide analog of thymidine which incorporates during the S phase of the cell cycle, whereas Dcx is a marker for neural progenitor cells (NPCs). Therefore, BrdU+/Dcx+ cells are a result of neurogenesis. For this assay, the rats received one injection of BrdU (Abcam, Cambridge, UK; 50 mg/kg) intraperitoneally every 12 hours for 5 days before day 120. The SC was then removed (1.0 cm caudal/rostral from the injury site). SC samples were perfused and fixed with 4% paraformaldehyde. Tissues were cut transversally with the cryostat into sequential serial sections (at 0, 2, 4, and 6 mm caudal and rostral from the epicenter). Slices were 40 μm thick, and a total of 48 sections per animal were counted and placed on slides using the free‐float method. Slides were washed twice for 10 minutes with PBS‐Triton (PBT) and incubated with ImmunoRetriever (Bio SB) for 60 minutes at 65°C. Afterward, slides were washed three times for 5 minutes with PBS and incubated for 30 minutes with 1N HCl at 37°C. When completed, they were incubated for 10 minutes with sodium borate 0.1 mol/L and washed three times with PBT. Unspecific binding sites were blocked with standard blocking solution using fetal bovine serum for 30 minutes. The primary antibodies against BrdU (Roche Diagnostics) (mouse IgG, 1:250) and Dcx (Santa Cruz Biotechnology) (goat IgG, 1:250) were incubated for 20 hours overnight. The next day, the slides were washed three times for 10 minutes with PBT and incubated with secondary antibodies (Invitrogen) (BrdU: donkey IgG; Dcx: rabbit IgG; all at 1:500) for 2 hours. Excess antibodies were removed by washing with PBT. Slides were counterstained with DAPI. Confocal images (40×; TIFF) were acquired using a Zeiss LSM 800 microscope. All areas were quantified as total number of cells in all SC samples by a blinded evaluator using cell counting software ImageJ 1.52a (NIH, V1.48, Bethesda). The total number of BrdU+/Dcx+ cells was obtained by averaging the total number of cells from three slides.^12^, ^13^

### 2.8 Enzyme‐linked immunosorbent assay (ELISA)

Eight weeks after PBS or A91 immunization, animals were euthanized with an overdose of sodium pentobarbital (80 mg/kg) and the SC samples were rapidly excised. Reagents, samples, and standards were prepared according to the instructions of rat IL‐4 ELISA Kit (Cell Applications), IL‐10 ELISA Kit (RayBiotech), BDNF ELISA Kit (Ray Biotech), GAP‐43 ELISA Kit (Cusabio), and TNF‐α ELISA Kit (OriGene). Briefly, 100 µL standard or 30 µg total protein samples were added to each well and incubated for 2 hours at 37°C. The liquid of each well was removed and not washed. Afterward, 100 μL of biotin antibody (1×) was added to each well and incubated for 1 hour at 37°C. Then, it was aspirated and washed three times. One hundred μl HRP avidin (1×) was added to each well and incubated for 1 hour at 37°C. Subsequently, it was aspirated and washed five times and 90 µL of TMB substrate was added to each well. Posteriorly, the samples were incubated and protected from light for 15‐30 minutes at 37°C. Finally, 50 μL Stop solution was added to each well and read at 450 nm within 5 minutes.

### 2.9 Statistical analysis

Data are displayed as mean ± standard deviation (SD), and statistical significance was established when *P* ≤ .05. GraphPad Prism 8.0 (GraphPad Software, Inc) was employed in statistical analysis. Data from the assessment of functional recovery were analyzed using an ANOVA for repeated measures with Bonferroni's post hoc test (BBB test) and Student's *t* test (von Frey test). Neurogenesis was analyzed by one‐way ANOVA followed by the Tukey‐Kramer post hoc test. ELISA was evaluated using a Student's *t* test.

## 3 RESULTS

### 3.1 Immunization with A91 peptide improves motor recovery after chronic SCI

**FIGURE 1:**
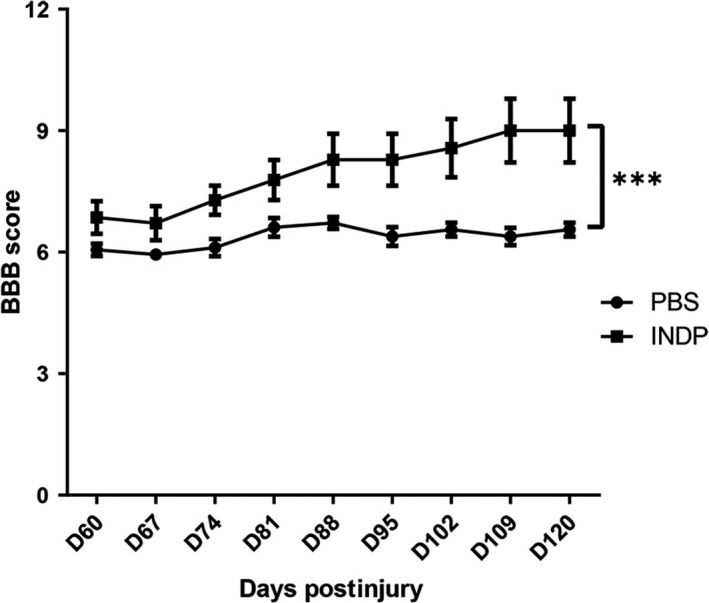
INDP induced a significant increase in motor recovery in the chronic stages of SCI. After the intervention, a significantly better motor recovery was observed in the INDP group when compared to the PBS‐immunized one. *Difference between the two treatment groups; *P* < .001, two‐way ANOVA for repeated measures with Bonferroni's post hoc test. Each point represents the mean ± SD of 9 rats

Eight weeks after PBS or A91 immunization, the group of rats submitted to INDP showed a significant increase in motor recovery (7.97 ± 0.87) when compared to those immunized with PBS alone (6.37 ± 0.27; *P* < .05, two‐way ANOVA for repeated measures with Bonferroni's post hoc test; Figure 1).

### 3.2 Sensitive function improves when immunizing with A91 peptide in the chronic stages of SCI

As the SCI and the subsequent inflammatory response trigger a nociceptive hypersensitivity reaction,^17^ we evaluated the development of mechanical hypersensitivity (MH). In this case, the lower is the damage, the less is the MH, and thereby, the withdrawal threshold is greater (the animal needs higher levels of pressure to stimulate withdrawal response). Therefore, hind paw MH was assessed by measuring withdrawal threshold mechanical stimulation with von Frey filaments.

**FIGURE 2:**
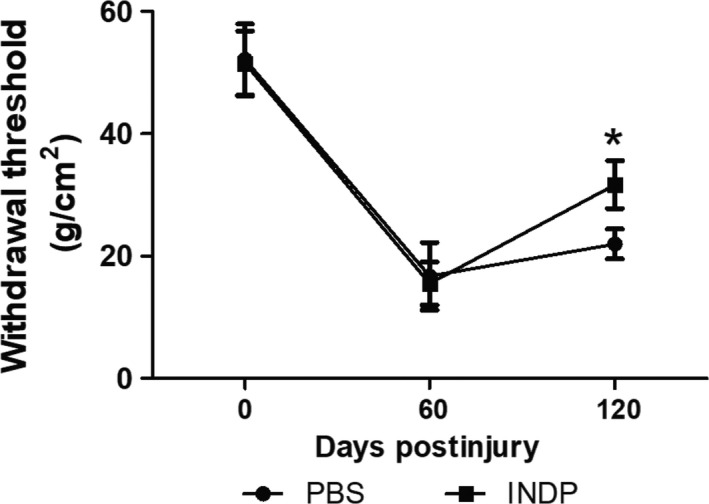
A91 immunization improved sensitivity function in the chronic stages of SCI. After the intervention, a significantly better withdrawal threshold was observed in the INDP group when compared to the PBS one. Each point represents the mean ± SD of 9 rats. *Different from the PBS group; *P* = .02, Student's *t* test

Evaluation of sensitivity function before therapeutic intervention demonstrated that the mechanical withdrawal threshold was similar in both groups (PBS: 52.19 ± 5.7, INDP: 51.53 ± 5.2, mean ± SD) and 60 (PBS: 16.71 ± 5.5, INDP: 15.52 ± 3.5) days after SCI. After 120 days, rats treated with INDP showed a significant increase (31.68 ± 3.9) in mechanical withdrawal threshold when compared to those with PBS immunization (21.97 ± 2.4; *P* = .02, Student's *t* test; Figure 2).

### 3.3 Neurogenesis is an active phenomenon in the chronic stages of SCI that can be increased by immunizing with A91 peptide

**FIGURE 3:**
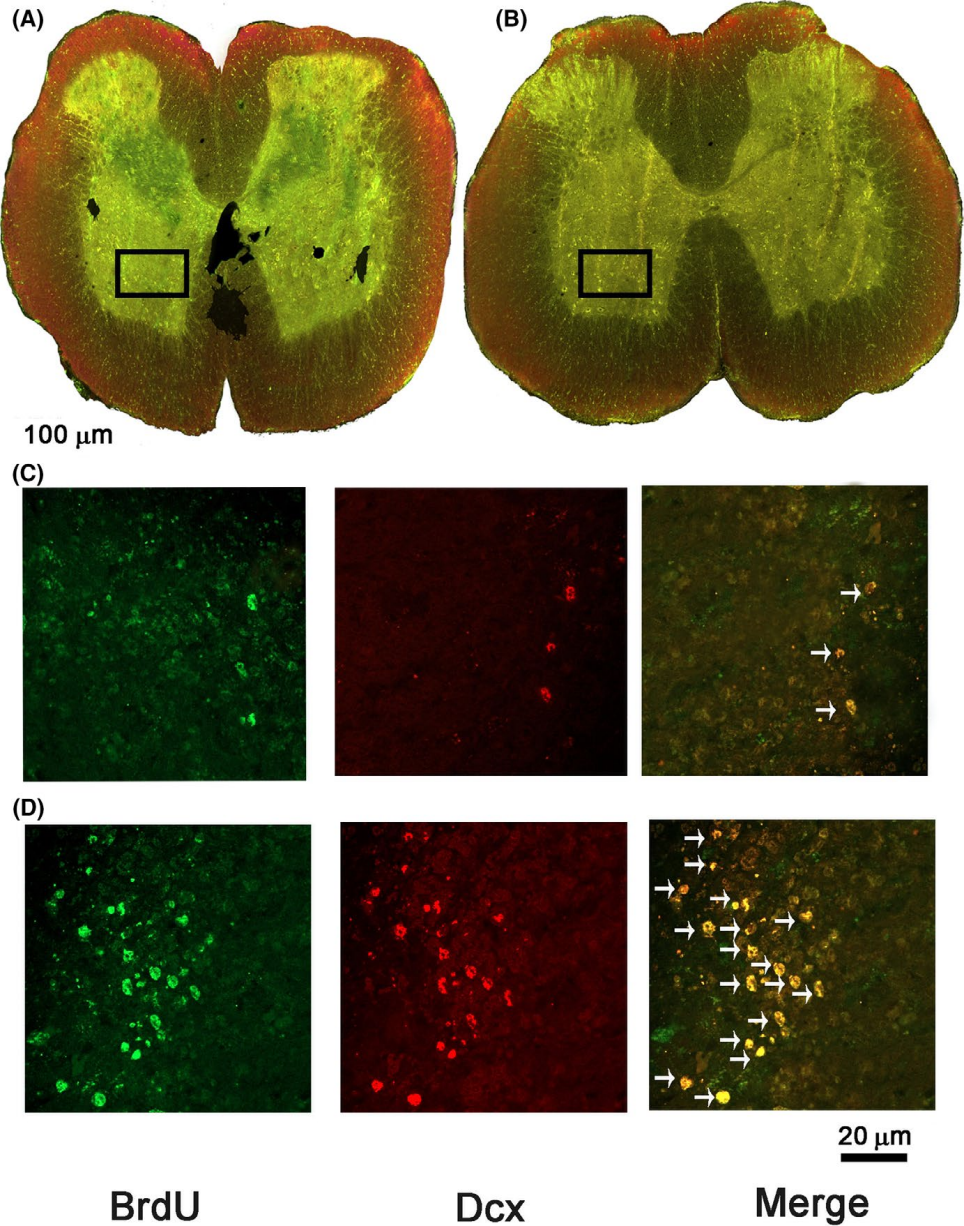
Representative microphotograph of BrdU+/DCX+ cells at the central canal, dorsal and ventral horns, and injury site of SC‐injured rats after therapeutic intervention. A and C correspond to the PBS, while B and D correspond to the INDP group. BrdU+ cells (green), Dcx+ cells (red), and BrdU+/Dcx+ cells (yellow). Black squares depict the amplified areas in C and D panels. Arrows indicate double‐labeled cells (neuroblasts). A greater number of double‐labeled cells were found in the INDP‐treated group

**FIGURE 4:**
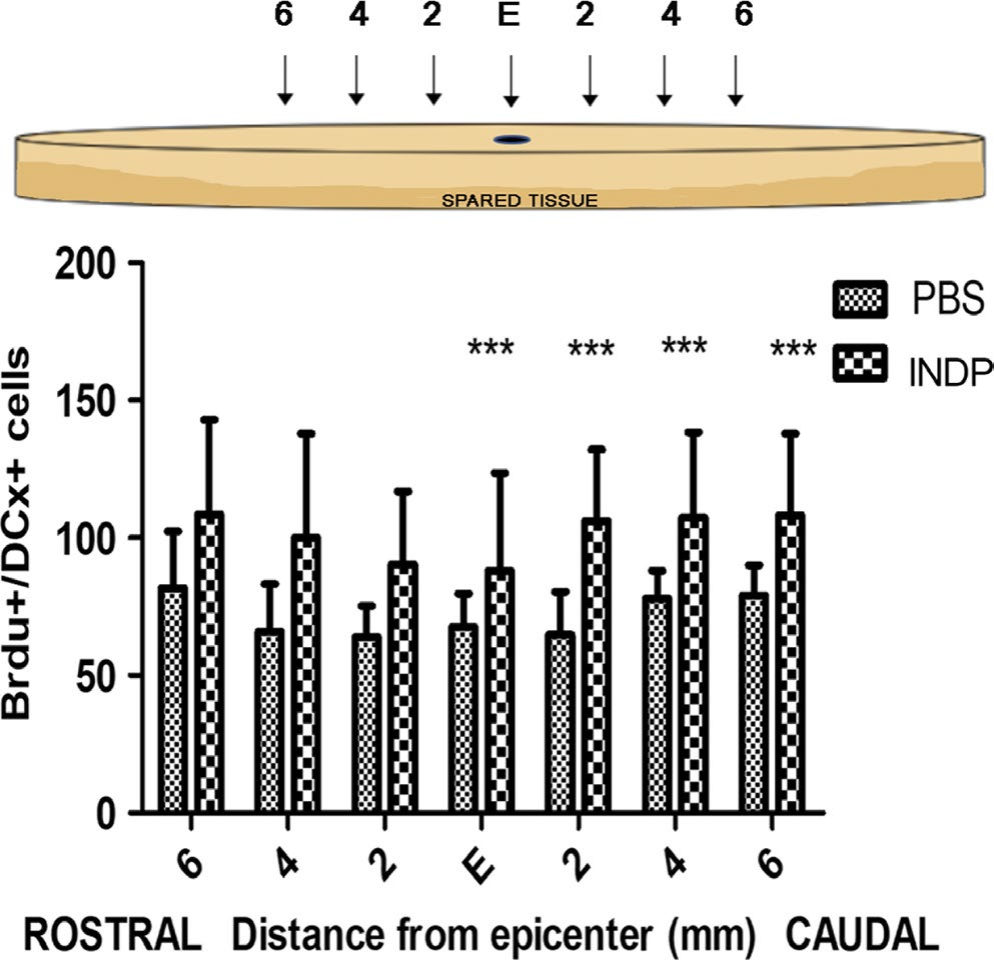
Number of BrdU+/DCX+ cells at the epicenter (E), rostral, and caudal stumps of the SC. The INDP group showed a significant increase in the total number of BrdU/DCX+‐labeled cells (neuroblasts) compared to the PBS group. Bars represent the mean ± SD of five rats. This is one representative graph of three determinations. *Different from PBS, *P* < .05; one‐way ANOVA followed by Tukey's test

In order to assess the number of NPCs in the area of injury, we labeled BrdU+/DCX+ cells at different sites from the epicenter of the lesion. We found an active neuroblast formation even in the SC of rats treated with PBS (see Figure 3A,C). Nevertheless, the amount of double‐positive cells was much higher in the group subjected to A91 immunization (Figure 3B,D). This effect was mainly observed at the caudal stump (at the central canal as well as at the dorsal and ventral horns), showing similar results from the epicenter 6 mm away from this site (Figure 4). The count of BrdU+/DCX+ cells revealed a higher number of NPCs in the group immunized with A91 peptide when compared to the one treated with PBS alone (*P* < .05, one‐way ANOVA followed by Tukey's test).

### 3.4 Immunization with A91 peptide induces a permissive microenvironment for neural restoration

The evaluation of gene expression (using qRT‐PCR) has shown that INDP induces a permissive microenvironment for neural restoration in the chronic stages of injury.^10^, ^11^ Based on these findings, we explored the induction of this microenvironment by directly analyzing the protein expression of antiinflammatory (IL‐4; IL‐10) and pro‐inflammatory (TNF‐α) cytokines. Additionally, we assessed the production of some regeneration‐associated proteins (BDNF and GAP‐43).

**FIGURE 5:**
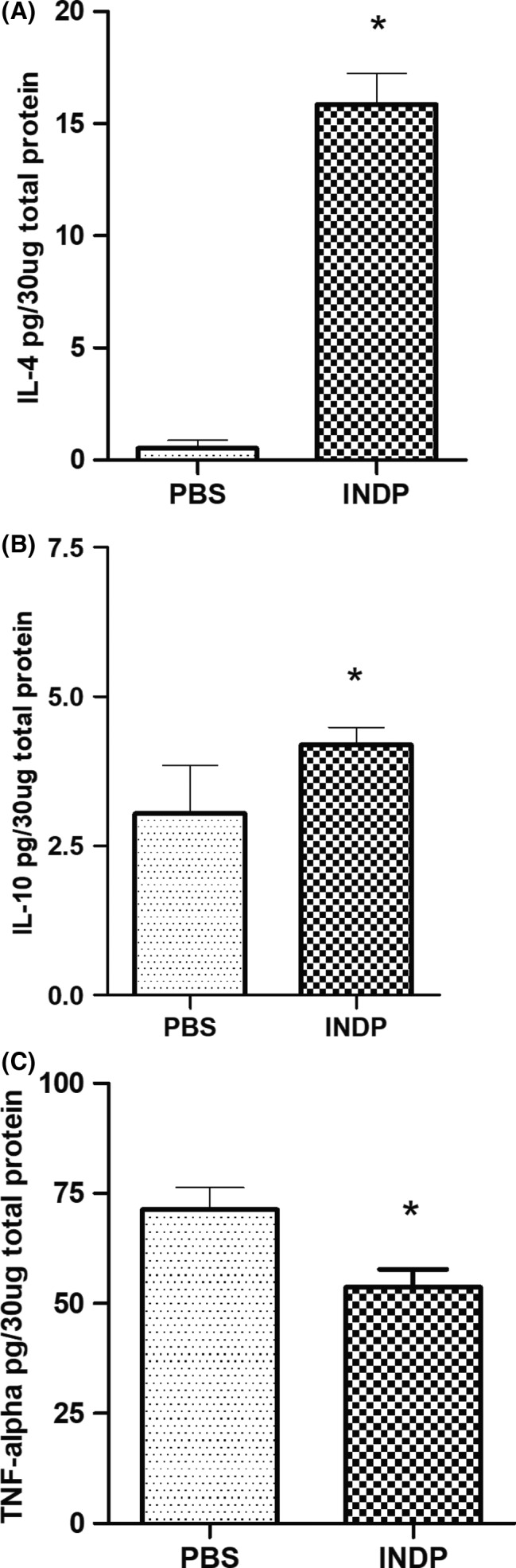
Concentration of antiinflammatory and pro‐inflammatory cytokines 120 d after SCI. The levels of IL‐4 (A) and IL‐10 (B) in the INDP group were significantly higher than those observed in PBS‐immunized rats. In contrast, the levels of TNF‐α (C) were significantly lower in rats treated with INDP compared to those immunized with PBS alone. Bars represent the mean ± SD of five rats. This is one representative graph of three determinations. *Different from PBS, *P* < .05; Student's *t* test

**FIGURE 6:**
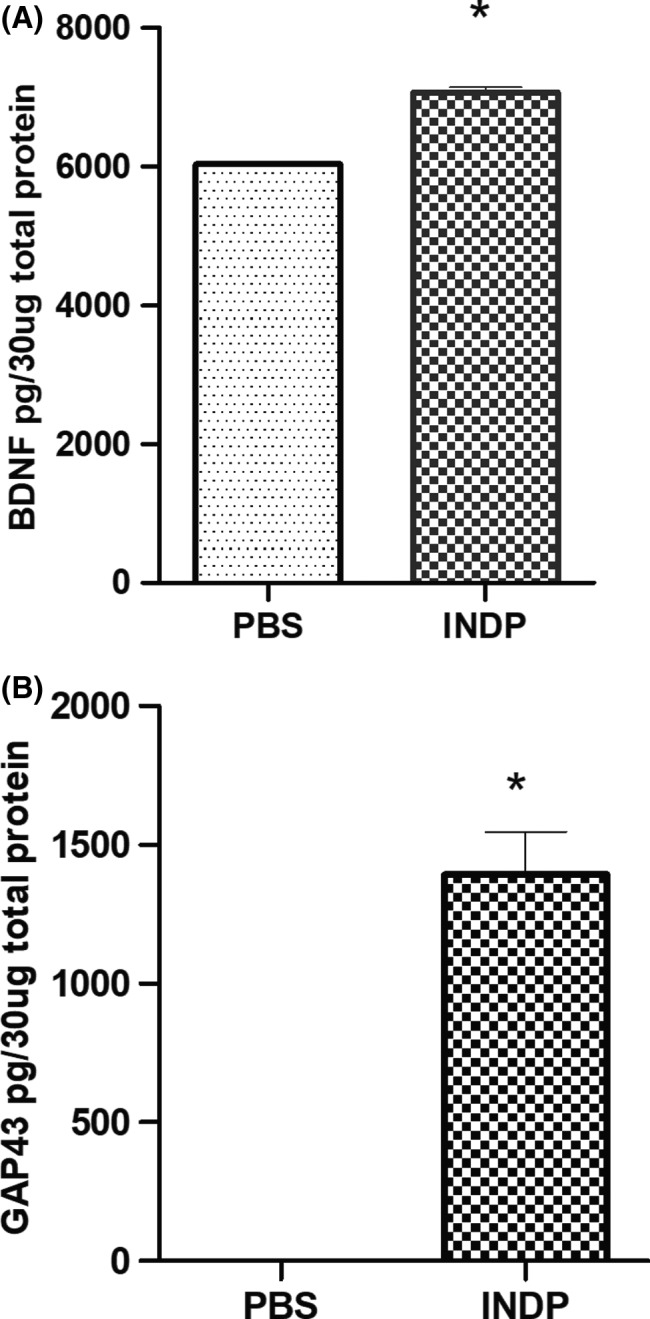
BDNF and GAP‐43 concentrations 120 d after SCI. The levels of BDNF (A) and GAP‐43 (B) in INDP‐treated rats were significantly higher than those observed in the group immunized with PBS alone. Bars represent the mean ± SD of five rats. This is one representative graph of three determinations. *Different from PBS, *P* < .05; Student's *t* test

Figures 5 and 6 show that INDP elicited a significant production of antiinflammatory and regeneration‐associated proteins in the chronic stages of SCI. Rats immunized with A91 peptide showed a significant increase in IL‐4 (15.85 ± 1.39; *P* = .0001, Student's *t* test; Figure 5A) and IL‐10 (4.18 ± 0.29, *P* = .0409, Student's *t* test; Figure 5B) when compared to PBS‐immunized ones (IL‐4:0.85 ± 0.36; IL‐10:3.03 ± 0.80). As expected, A91 immunization induced a significant decrease in TNF‐α (53.62 ± 4.051) when compared to the group immunized with PBS alone (71.19 ± 5.02, *P* = .0092, Student's *t* test; Figure 5C). In regard to BDNF (Figure 6A) and GAP‐43 (Figure 6B), a significant increase in these molecules was also observed in A91‐immunized rats (BDNF: 7068 ± 73.73, *P* = .0001, Student's *t* test; GAP‐43:1392 ± 155.50, *P* = .0001 Student's *t* test) when compared to those immunized with PBS alone (BDNF: 6034 ± 15.73, *P* = .0001, Student's *t* test; GAP‐43:0.85 ± 1.47, *P* = .0001 Student's *t* test).

## 4 DISCUSSION

Previous studies in our laboratory have shown that INDP induces axonal regeneration and functional recovery in the chronic phase of SCI.^10^ On this basis, we tried to prove whether INDP is also capable of inducing neurogenesis. As shown in our results, after SCI there is an important development of neuroblasts, even in chronic stages of injury particularly at the central canal but also at the dorsal and ventral horns. These newly formed cells increased significantly when rats were subjected to INDP.

There are well‐defined neurogenic areas in the adult central nervous system (CNS): the subventricular and subgranular zones (SVZ and SGZ, respectively).^18^, ^19^ For a long time, it was thought that neurogenesis took place only in these two areas. Nevertheless, recent reports suggest that neurogenesis in the adult state can be found in other regions of the CNS, such as the amygdala, neocortex,^20^ cerebellum, striatum, and substantia nigra.^21^, ^22^ Additionally, neurogenesis is also amplified after mechanical damage due to traumatic brain injury^23^ or SCI.^24^

Regarding the latter, induction of neurogenesis after the injury has already been documented. For instance, previous studies have shown that SCI activates neurogenic processes, especially at acute phases^25^; however, little is known about these events in the chronic stages of SCI. In this study, we demonstrate that neurogenesis continues to be an active process even 120 days after SCI. This is an important finding as it reveals that even at chronic stages of injury; there are mechanisms that aim to restore neural tissue. The presence of neurogenesis long after the injury is a totally understandable event as ependymal cells—a potential origin of endogenous NPCs—are actively proliferating even several weeks after SCI.^25^, ^26^, ^27^ Therefore, it is expected to find newly formed neurons derived from ependymal cells in the chronic stages of injury.

Neurogenesis in chronic SCI is a topic that deserves to be further studied since it represents an important process being activated—even long after injury—as an effort to restore neural connections. Unfortunately, under pathophysiological conditions (with no therapeutic intervention) this restoration attempt does not contribute to a functional neurological recovery, as was shown in the present study. For that reason, neural restoration necessarily requires to be boosted with additional therapeutic approaches that in some way improve events such as neurogenesis, inducing then functional recovery after a SCI.

In the present investigation, we intended to boost neurogenesis using INDP, a therapeutic approach that has previously shown to increase the formation of neuroblasts after CNS disorders, such as stroke.^13^, ^28^ Our results show that INDP is capable of increasing neurogenesis. This increase was significantly associated with better sensory and motor recovery. Previous studies have shown that INDP induces both motor and sensitive restoration in both acute^29^, ^30^ and chronic^10^, ^11^ stages of injury. These beneficial effects could be the result of the increase in antiinflammatory and regeneration‐associated proteins as well as the reduction of pro‐inflammatory molecules which contribute inducing a favorable microenvironment for neurogenic processes. There was a significant increase in IL‐4 and IL‐10 which are strongly related to neuroprotective and regenerative actions as well as with axon elongation.^31^

Motor and sensory recovery could be explained by the appearance of newly descending fibers. Previous studies demonstrate that INDP increased the number of serotonergic (5‐HT‐positive) and catecholaminergic (TH‐positive) axons, especially at the caudal stump of the SC.^10^

On the other hand, the increase in 5‐HT and TH‐positive axons specifically at the caudal stump of the injury—a consistent finding of previous studies—^9^, ^10^ also explains, at least in part, the significant increase in neuroblasts at the caudal stump of SCI. In fact, serotonin and catecholamines have also been suggested as possible neurogenic factors.^32^, ^33^, ^34^, ^35^, ^36^ The reason why the restorative effects are more directed to the caudal stump is an issue that should be addressed in future research; for now, we could only speculate that it is the result of a restorative response to the neural disconnection that is being boosted by INDP, for then promoting a better permissive microenvironment for neural restoration. Further studies should be designed to elucidate this interesting topic.

The neurogenic effect of INDP, as well as the formation of new fibers, could reinforce the formation of connections between interneurons at the ventral and dorsal horns, thus providing the ability to convey efficient information through afferent or efferent fibers.^37^, ^38^

Neurogenesis after SCI is a phenomenon that has already been documented. Almost two million new cells are produced at the site of injury.^39^ Moreover, the induction of proliferation, migration, and differentiation of NPCs toward NeuN+ mature neurons after SCI in mice has been described as well.^40^ Nevertheless, the origin of these cells is still unknown and has not been well documented. Currently, there is not much information about the existence of any neurogenic niche in the SC. A particular emphasis has been placed on the ependymal channel since proliferation, migration, and differentiation of ependymal cells into NPCs have been reported in SC regions after injury.^26^, ^41^ Other studies have shown that ependymal tanycytes have neurogenic potential.^42^ Therefore, the ependymal channel is emerging as the main possible source of neurogenesis; however, neuroblasts could also derive from meningeal cells^43^ or even from very distant places like the hippocampus.^44^ This is a topic that should be further addressed to gain new insights into NPCs origin following A91 therapy in chronic SCI.

On the other hand, the mechanisms by which INDP induces neurogenesis are also a topic for further analysis. After SCI, INDP induces the activation of Th2‐lymphocytes, a phenotype that releases high concentrations of IL‐4 and IL‐10^10^, ^45^ which have been strongly associated with the induction of neurogenesis.^28^, ^46^, ^47^, ^48^, ^49^ In the present study, we show that INDP effectively induces the release of high concentrations of IL‐4 and IL‐10. In addition, INDP decreases pro‐inflammatory cytokines as well. All of these findings have already been reported in both acute and chronic phases of injury and have been proposed as important factors that contribute to the formation of a permissive microenvironment, which favors neural tissue restoration.^10^, ^45^

In order to better understand the way by which INDP boosts neurogenesis, we also determined the concentrations of BDNF and GAP‐43, two molecules strongly associated with neurogenesis.^50^ Regarding this, we found a significant increase in both molecules after INDP treatment. Several studies have previously shown that BDNF/TrkB signaling pathway is strongly involved in the induction of neurogenesis.^50^, ^51^ Similarly, GAP‐43 upregulation contributes to neurogenesis as it is involved in the orientation of cell division and is also required for neurons' maturity.^52^, ^53^, ^54^, ^55^ In this regard, it is important to remark that GAP‐43 concentrations were extremely higher than those observed in PBS‐treated rats. This finding supports the results of previous studies^9^, ^10^ and strongly suggests that GAP‐43 is one of the primary mediators of the restorative effects of INDP. However, future studies should address this topic.

INDP is emerging as a novel neurogenic strategy that could potentially be used for several CNS pathologies, in addition to SCI. For instance, previous studies have shown the neurogenic effect of INDP administration following cerebral ischemia.^13^ Given the similar pathophysiological components between traumatic brain injury and SCI, it is plausible to envision the utility of INDP in such diseases. This exciting possibility warrants further investigation.

The results of this study show that neurogenesis is a phenomenon that is still activated even several weeks after injury and not only in the acute phase. In addition, we demonstrate that INDP is capable of increasing neurogenesis in the chronic stages of injury. This beneficial effect is associated with a better motor and sensitivity recovery. Further studies are needed in order to better understand the origin, dynamics, and functionality of this neurogenic phenomenon induced by INDP after SCI. Additionally, it is important to see that this therapeutic strategy could also be beneficial in cases of other trauma‐related disorders such as traumatic brain injury.

## CONFLICT OF INTEREST

The authors declare that they have no competing interests. The funders had no role in study design, data collection and analysis, decision to publish, or preparation of the manuscript.
